# Systemic gene delivery to the central nervous system using Adeno-associated virus

**DOI:** 10.3389/fnmol.2014.00050

**Published:** 2014-06-02

**Authors:** Mathieu Bourdenx, Nathalie Dutheil, Erwan Bezard, Benjamin Dehay

**Affiliations:** ^1^Institut des Maladies Neurodégénératives, UMR 5293, Université de BordeauxBordeaux, France; ^2^CNRS, Institut des Maladies Neurodégénératives, UMR 5293Bordeaux, France

**Keywords:** gene therapy, AAV, neurological disorders, neurodegenerative diseases, systemic delivery

## Abstract

Adeno-associated virus (AAV)-mediated gene delivery has emerged as an effective and safe tool for both preclinical and clinical studies of neurological disorders. The recent discovery that several serotypes are able to cross the blood–brain barrier when administered systemically has been a real breakthrough in the field of neurodegenerative diseases. Widespread transgene expression after systemic injection could spark interest as a therapeutic approach. Such strategy will avoid invasive brain surgery and allow non-focal gene therapy promising for CNS diseases affecting large portion of the brain. Here, we will review the recent results achieved through different systemic routes of injection generated in the last decade using systemic AAV-mediated delivery and propose a brief assessment of their values. In particular, we emphasize how the methods used for virus engineering could improve brain transduction after peripheral delivery.

## INTRODUCTION

In the last decade, adeno-associated-virus (AAV)-mediated gene delivery has emerged as an effective and safe tool for both preclinical and clinical studies of neurological disorders (Bartus et al., 2013; Weinberg et al., 2013; Ojala et al., 2014). Currently, AAV is the most widely used vector for clinical trials for neurological disorders (gene therapy database can be found at: http://www.abedia.com/wiley/index.html). To date, no adverse effects linked to the use of this vector have ever been reported from clinical trials. Adeno-associated virus is a non-pathogenic dependovirus from the *parvoviridae* family requiring helper functions from other viruses, such as adenovirus or herpes simplex virus, to fulfill its life cycle (Dayton et al., 2012). The wild-type (WT) AAV is characterized by a single-stranded DNA (ssDNA) genome, with inverted terminal repeats (ITR) at both ends, of approximately 5 kb surrounded by a capsid. Advances in process development have made AAV production fast, reliable, highly pure, and affordable.

The first recombinant AAVs of serotype 2 (rAAV2) have been generated in the 1980s after the removal of 96% of the viral genome (Samulski et al., 1982; Hermonat and Muzyczka, 1984; McLaughlin et al., 1988). Only the two ITRs containing replication origin and encapsidation signal remained making it a safe, non-replicative virus. Further studies allowed the production of high-titer rAAV batches in the absence of WT virus and adenovirus (Ferrari et al., 1997; Grimm et al., 1998; Xiao et al., 1998). Moreover, AAVs have been reported to transduce both dividing and non-dividing cells as well as a wide range of tissue while remaining being poorly immunogenic, making it an ideal candidate for gene delivery to the CNS (Weinberg et al., 2013). Taking advantage of progresses made in rAAV production, the first clinical trials for neurological disorders, such as Parkinson’s disease, using rAAV2 vectors opened a new era (Kaplitt et al., 2007; Marks et al., 2010; Bartus et al., 2013).

Phylogenic studies of capsid protein sequence from human and non-human primate (NHP) tissue allowed the characterization of distinct clades or families (Gao et al., 2004). Recently, 12 of these AAV serotypes have been engineered into rAAV (Porras et al., 2014). Thanks to their different capsid composition, the multiple serotypes exhibit distinct transduction profiles compared to rAAV2 (Vandenberghe et al., 2008). The ability of AAV2 ITRs to package any of the serotype capsids allows efficacy comparison between serotypes *in vivo* (Rabinowitz et al., 2002). To date, while rAAV2 is the most widely used in clinical trials, most of the other serotypes have shown an enhanced ability to transduce neurons in experimental studies (Davidson et al., 2000; Taymans et al., 2007; Tarantal and Lee, 2010).

It is difficult to define the best serotype for intraparenchymal CNS injections since species and cerebral structures have been shown to influence transduction success (Taymans et al., 2007; Korecka et al., 2011; Weinberg et al., 2013). At least one study reported that overexpression of the microtubule-associated protein tau peaked earlier when mediated by rAAV9 or rAAVrh10 than by rAAV2 or rAAV8 despite their 2.1-fold lower dose of virus genome (vg; Klein et al., 2008).

Among the different serotypes, only a few have been shown to efficiently cross the blood–brain barrier (BBB; Zhang et al., 2011). The BBB deprives the brain of > 98% of neurotherapeutic compounds (Pardridge, 2002). In this context, gene therapy has been proposed as a means of crossing the BBB (Foust et al., 2008). Widespread transgene expression after systemic injection, although challenging, could be of interest for therapeutic approaches. Such a strategy would avoid invasive brain surgery and allow promising non-focal gene therapy for CNS diseases such as lysosomal storage disorders (LSDs) or Alzheimer’s disease, which knowingly affect large part of the brain.

## WIDESPREAD BRAIN TRANSDUCTION AFTER SYSTEMIC INJECTION

As this field of research is booming, we review here the recent results achieved through different systemic routes of injection, such as intramyocardialy, intramuscularly, and intravascularly, generated in the last decade using systemic AAV-mediated delivery. In addition, we propose a brief assessment of their values.

Successful gene therapies for brain diseases require a widespread distribution and magnitude of transgene expression throughout the brain. Several studies reported an efficient gene delivery to motor neurons after retrograde transport of viral particles injected intramuscularly (i.m.; Kaspar, 2003; Miller et al., 2005). This strategy enabled the delay of disease onset and the increase of lifespan in a mouse model of amyotrophic lateral sclerosis (ALS; Kaspar, 2003). However, targeting specific brain areas such as the cerebral cortex would have required repeated injections, thus preventing the clinical application of such an approach (Foust et al., 2008). Conversely, efficient brain transduction after single systemic injection of AAV particles has been recently reported in several species such as mice, rats, cats, and monkeys (**Table 1**; Foust et al., 2008; Duque et al., 2009; Wang et al., 2010; Dehay et al., 2012). Foust et al. (2008) demonstrated a greater neuronal tropism after injection in neonatal mice through the facial vein while injection into adult mice through the tail vein led to glial (mostly astrocytic) transduction. However, Duque et al. (2009) reported up to 28% of transduction of cervical spinal cord in adult mice after intravascular (i.v.) injection suggesting that this route of injection might be effective for brain transduction in adult animals, even though the transduction efficiency was variable. Again, systemic injection of rAAV9 to neonate cats also showed better transduction efficiency than in adult animals (Duque et al., 2009). Intravenous administration of rAAV9 to neonatal rats showed up to 78% of transduction of motor neurons of the spinal cord associated with widespread CNS transduction (Wang et al., 2010). Altogether, these studies suggest that injection in neonatal animals is more successful compared to injection in adult animals for widespread brain transduction. This strategy has been successfully applied to a spinal muscular atrophy (SMA) mouse model (Foust et al., 2010). In this pioneer work, the group of Brian Kaspar injected rAAV9 expressing the survival motor neuron (SMN) protein at postnatal day 1 (P1) into SMA animals allowing rescued motor function and increased lifespan (Foust et al., 2010). Interestingly, treatment at postnatal day 5 partially rescued the phenotype while treatment at postnatal day 10 had barely any effect (Foust et al., 2010). The decreased effect of treatment over time can be correlated with the increased glial transduction. To date, it is not yet fully understood why neuronal transduction in adult brains is not as powerful as in neonatal animals. Several factors have been proposed such as differences in extracellular matrix composition, neuron-to-glia ratio or BBB maturity although these hypotheses remain controversial (Lowenstein, 2009; Saunders et al., 2009). Recently, the group of Andrea Ballabio used a combined approach with both intracerebral ventricle injection and systemic injection of rAAV9 to achieve whole-body transduction in a multiple sulfatase deficiency (MSD) mouse model (Spampanato et al., 2011). Although the combined approach reverses the phenotype of this severe LSD, the intracerebral ventricle injection explained mainly the brain transduction while the systemic injection induced most of the peripheral transduction (Spampanato et al., 2011). Several studies confirmed the reproducibility of rAAV9 intravenous injection with a dose-dependent CNS transduction in neonatal mice associated with a sustained expression of up to 18 months post-injection (Gray et al., 2011b; Miyake et al., 2011). While most studies used rAAV9, several other serotypes have also been shown to cross the BBB and induce a robust CNS transduction as well (Zincarelli et al., 2008; Zhang et al., 2009). Among them, rAAVrh10 appeared at least as efficient as rAAV9 in CNS transduction after i.v. injection into neonatal mice (Zhang et al., 2009).

**Table 1:** Summary of literature reports using AAV vectors for systemic gene delivery to the CNS in mammals.

Study	Serotype	Virus type	Reporter	Administration	Animal	Titer	Remarks
Miyake et al. (2011)	1, 8, 9, 10	SS	GFP	i.v. (jugular vein)	Mice (PI, P5, P14, P42)	1.5 × l0^11 ^vg/pup	rAAV9 was the most efficient
						1.5 × 10^12^ vg/adult	Transduction efficiency decrease over time
Duque et al. (2009)	9	SC	GFP	i.p i.m. (triceps and gastrocnemius) i.v. (temporal vein)	Mice (PI and 8 weeks) Cats (P2 and 7 weeks)	i.p: 10^10^ vg/mouse i.m.: 10^9^ vg/mouse i.v.: 10^11^ vg/kg	i.v. route of delivery was superior to the other
Zhang et al. (2011)	1, 2, 5, 6, 6.2, 7, 9, rhlO, rh39, rh43	SC	eGFP	i.v. (temporal vein)	Mice (PI)	4 × 10^11^	9, rhlO, rh39, rh43 were the most efficient
Rahim et al. (2011)	9	SS and SC	GFP	*in utero* (vitelline vessels) i.v. (temporal vein)	Mice (E15 and PI)	2 × lO^11^ vg/embryo 4 × l0^11^ vg/pup	*In utero* delivery was more efficient than PI
Mattar et al. (2012)	9	SC	eGFP	*In utero*	Cynomolgus macaques (E140)	3 × 10^11^ vg/kg	Transduction was mostly neuronal, up to 97% in the cerebellum
Zincarelli et al. (2008)	1, 2, 3, 4, 5, 6, 7, 8, 9	SS	Luciferase	i.v. (tail vein)	Mice (8–10 weeks)	1 × 10^11^	Luciferase activity in the brain was detected only after rAAV8 or -9 injection
Dehay et al. (2012)	9	SC	GFP	i.v. (saphenous vein)	Rhesus macaques	1.33 × 10^15^ vg/kg	Post-natal developed structures were more transduced than the others
Bevan et al. (2011)	9	SC	GFP	i.v. (saphenous vein)	Cynomolgus Macaques (P1–P90, 3-year-old)	l–3 × l0^1^^4^ vg/kg to Pl–P90 animals 2.7 × 10^11^ vg/kg to adult animals	Injection to young animal was more powerful compared to injection to adult (partially attributable to the dose)
Samaranch et al. (2012)	9	SS and SC	GFP	i.v. (carotid artery) i.cm.	Cynomolgus and Rhesus macaques	3 × 10^13^ vg/kg for i.v. 1.8 × l0^3^ vg/kg for	Similar pattern for both route i.c.m. injection led to stronger expression than i.v. injection
Gray et al. (2011b)	9	SS and SC	GFP	Mice: i.v. (tail vein)	Mice (8–12 weeks old)	Mice: up to 8 × 10^13^ vg/kg	Dose-dependent transgene expression
				Monkey: i.v. (saphenous vein)	Rhesus monkeys (3–4-year-old)	Monkey: l × 10^11^ vg/kg	Shift toward glial transduction was more pronounced in monkeys
Foust et al. (2008)	9	SC	GFP	Neonates: i.v. (facial vein) Adults: i.v. (tail vein)	Mice (PI and 10 weeks)	4 × 10^11^ vg/animal	Neuronal transduction in neonates Astrocytic transduction in adults

*SS, single stranded; SC, self-complementary; GFP, green fluorescent protein; eGFP, enhanced GFP; i.v., intravascular; i.m., intramuscular; i.p, intraperitoneal; i.c.m., intra *cisterna magna*; P1, postnatal day 1; E15, embryonic day 15; vg, virus genome.*

Consistent with mouse, rat, and cat studies, efficient gene delivery and brain transduction has been reported after systemic injection of rAAV in macaque monkeys (Bevan et al., 2011; Gray et al., 2011b; Dehay et al., 2012; Mattar et al., 2012; Samaranch et al., 2012). Even though some methodological discrepancies between studies prevent a clear comparison of the results, some conclusions can be achieved. Systemic administration of rAAV9 to adult monkeys induced mostly glial transduction (Bevan et al., 2011; Gray et al., 2011b; Samaranch et al., 2012) while injection in neonate animals induced neuronal transduction (Dehay et al., 2012; Mattar et al., 2012). Another concern in this field of research involves anti-AAV antibodies, which are commonly present in both non-human primate and humans (Boutin et al., 2010; Calcedo et al., 2011; Gray et al., 2011b; Samaranch et al., 2012). Such antibodies might prevent efficient brain transduction and might explain the weaker transduction into adult animals since anti-AAV antibody concentration has been reported to increase with time suggesting that “the sooner the better” is the *credo* for systemic injection in NHP (Calcedo et al., 2011). However, with regard to these discrepancies between studies, a high-titer dose of viral particles appeared to be the common denominator for monkey injection (Bevan et al., 2010; Gray et al., 2011b; Dehay et al., 2012; Mattar et al., 2012; Samaranch et al., 2012).

These critical points in mind, gene delivery to fetuses could be clinically relevant for early-onset diseases associated with neurodegeneration and early death in childhood such as Type II Gaucher disease (GD). In this particular disorder, brain pathology can be detected *in utero* and death occurs within the two first years of age (Sidransky et al., 2009). Correspondingly, two studies administered rAAV9–GFP to fetal mice or monkeys and reported a robust central and peripheral transduction (Rahim et al., 2011; Mattar et al., 2012). Both studies reported a strong transduction of neuronal cells compared to astrocytes, surpassing that of neonatal injection (Rahim et al., 2011; Mattar et al., 2012). Even though this strategy has not been applied yet to a disease model such as Type II GD, *in utero* delivery of a therapeutic gene might improve phenotype of early-onset diseases.

Several routes of administration have been tested to obtain widespread brain transduction. As mentioned earlier, large brain transduction has been reported after intravenous injections of rAAV (Foust et al., 2010; Dehay et al., 2012). Interestingly, a single intracardiac injection of rAAV to adult mice has been reported to preferentially transduce glial cells and to reduce amyloid-β peptide levels in the brain (Iida et al., 2013; Iwata et al., 2013). Once again, the strong glial transduction might be due to the time of injection. More invasive protocols such as intrathecal intra-*cisterna magna* injections of rAAV9 have been reported to efficiently transduce cerebral tissue in both NHPs and pigs (Federici et al., 2012; Samaranch et al., 2012). Intra-*cisterna magna* and other intrathecal injections reported a stronger transduction compared to i.v., i.m., s.c., and sciatic nerve injections (Towne et al., 2009; Samaranch et al., 2012). Although interesting, this approach could not circumvent the antibodies issue in NHP (and hence man; Samaranch et al., 2012). Finally, some groups used intranasal administration of rAAV and reported an efficient transduction mostly in olfactory bulbs and lungs (Zhang et al., 2003; Wolf et al., 2012).

Widespread brain transduction after systemic injection of rAAV might have two distinct applications. First, AAV-mediated expression of a given pathogenic protein in the whole brain could be an alternative modeling system to the classic toxic-based or local injection models of non-focal neurological disorders. Moreover, such strategy might allow global CNS transduction of animals such as rats or NHP, which remains difficult to achieve by classical transgenesis. Second, widespread silencing of a pathogenic protein or expression of a rescue protein might be useful for therapeutic interventions. However, gene therapy for neurodegenerative disorders, which are mainly adult-onset diseases, would ideally occur in adult, unless familial diseases or cases of otherwise sporadic diseases are targeted. Even if glial transduction might have clinical relevance for diseases such as amyotrophic lateral sclerosis (ALS) or Parkinson’s disease (Nagai et al., 2007; Colangelo et al., 2014), a strong cell-type-specific transduction is mandatory for clinical application of systemic gene delivery via rAAV. Moreover, the large titers of virus used in NHPs suggest that even higher titers should be used in humans. These limitations stress the need for more powerful and precise vectors as well as an optimized route of administration.

## CONTROL OF TRANSGENE EXPRESSION, CELL SPECIFICITY, AND VECTOR OPTIMIZATION

### VIRUS GENOME TUNING

**FIGURE 1:**
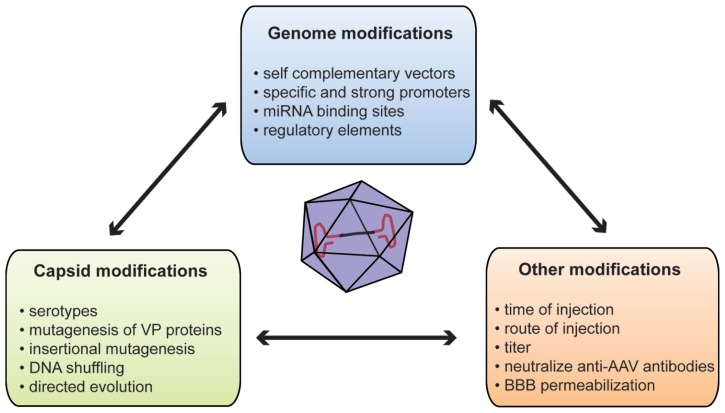
**Summary of methods used to improve gene delivery after systemic injection of AAVs.** AAV, adeno-associated virus; BBB, blood–brain barrier; miRNA, microRNA; VP, viral particle protein.

To overcome these limitations, several methods have been developed to improve brain transduction after systemic injection (**Figure 1**). The WT AAV genome is packaged as a linear ssDNA with ITRs at both ends. Host-cell-mediated synthesis of the second strand of the AAV genome has been shown to be the rate-limiting step of transduction with rAAV (Ferrari et al., 1996). Thus, McCarty et al. (2003) deleted the terminal resolution site from one ITR to generate so-called self-complementary vectors (scAAV) with a 10 to 50-fold stronger gene expression than single-stranded vectors. However, such an increase in gene expression leads to the loss of half of the packaging capacity of the vector (2150 bp for scAAV2; McCarty et al., 2003). Such increased expression has been reported in a systemic gene delivery study where the number of reporter-positive cells after ssAAV9 injection was similar to that obtained with a 20-fold lower dose of scAAV9 (Gray et al., 2011b).

One of the main concerns of systemic gene delivery via i.v. injection of rAAV might be off targets. Several studies reported transduction of peripheral organs, such as skeletal muscle, heart, pancreas, or antigen-presenting cells after systemic injections (Zincarelli et al., 2008; Rahim et al., 2011; Mattar et al., 2012). Although it can be of significance for diseases with peripheral and central components such as LSD, this can raise the apprehension of unwanted protein overexpression external to the CNS potentially eliciting toxic responses (Xie et al., 2011). At the vector genome level, two distinct but complimentary strategies could be used to specify gene expression: the first is based on cell-type-specific promoters restricting transgene expression to certain cell subpopulations; conversely the second involves the repression of transgene expression in unwanted cells or organs. In this context, promoter choice is critical since it can determine the strength or specificity of expression. Most intracerebral AAV injections used a cell-type-specific promoter such as the synapsin promoter to restrict expression to neurons (Decressac et al., 2012; Engeln et al., 2013). However, systemic gene delivery studies mostly use strong and ubiquitous promoters including the cytomegalovirus (CMV) promoter or the truncated chicken beta actin (CBA) promoter (Dalkara et al., 2011; Mattar et al., 2012). The restricted packaging capacity of ssAAV and even more of self-complementary vectors stresses the need for minimal and strong promoters. To fulfill this need, Gray et al. (2011a) developed a hybrid CBA (CBh) promoter of 800 base pairs (bp) allowing more stable, longer, and stronger expression compared to CMV or CBA promoters. This expression can be further enhanced by the use of 5′ or 3′ untranslated regions (UTR). Of note, the woodchuck hepatitis virus post-transcriptional response element (WPRE) has been shown to improve brain transduction after intracerebral injection (Hermening et al., 2006; Decressac et al., 2012). This increased transduction comes with a cost of 600 bp of packaging size (Hermening et al., 2006). Rahim et al. (2011) compared brain and eye expression after *in utero* injection of scAAV9 without WPRE element versus ssAAV9 carrying the WPRE sequence. They observed that the scAAV9 vector including the WPRE sequence is more efficient than an scAAV9 vector deprived of WPRE. Subpopulation-specific neuronal promoters are often long DNA sequences with regulatory elements. For instance, the full mouse tyrosine hydroxylase promoter that controls expression in dopaminergic neurons is 7.5 kb making it too voluminous for use in an rAAV (Iwata et al., 1992). In this context, the use of tissue-specific microRNAs (miRNAs)-binding site in the AAV genome could overcome this promoter size limitation by repressing expression in tissue that express the miRNAs (Xie et al., 2011). Such a strategy might be of interest for systemic gene delivery. Indeed, the incorporation of three copies of miRNA122-binding site or miRNA1-binding site in the rAAV genome dramatically decreases transduction in liver or heart, respectively (Xie et al., 2011). Reduction of hepatic transgene expression after systemic gene delivery might be an added benefit as liver is a key target of AAV vectors.

### CAPSID TUNING

Virus capsid is the other obvious target to engineer to improve or specify transgene expression. A WT AAV capsid comprises three structural Cap proteins: VP-1, -2, and -3 with a ratio of 1:1:10. The capsid is the primary interface between virus and host cell, mediating vector binding to cell surface receptors (Wu et al., 2006). Moreover, the capsid but not the ITR sequence influences cell and tissue tropism (Grimm et al., 2008; Vandenberghe et al., 2008). The propensity of certain AAVs serotypes to bypass anatomical barriers is directly related to their capsid composition since only several serotypes with identical genome are able to efficiently cross the BBB (Zhang et al., 2011). Glycans with terminal β-galactose linkages have been recently identified as the primary receptor for AAV9 (Bell et al., 2011; Shen et al., 2011). This unique feature of serotype 9 might be related to its ability to cross the BBB. The 37/67-kDa laminin receptor has been identified as co-receptor for AAVs of serotypes 2, 3, 8, and 9; however, its involvement in BBB crossing has not been determined yet (Akache et al., 2006).

Capsid engineering can be designed to produce new AAV variants by (i) mutagenesis of VP proteins, (ii) incorporation of specific peptide ligand at the virus surface or (iii) directed evolution (Gray and Samulski, 2011). One example of capsid mutagenesis is tyrosine substitution. Zhong and colleagues reported that rAAV2 capsid could be phosphorylated on surface-exposed tyrosines leading to ubiquitinylation and degradation of viral particles (Zhong et al., 2008). Mutagenesis of one or more of the seven surface-exposed tyrosine residues to phenylalanine (Y–F) has been reported to reduce proteasomal degradation of viral particles and therefore enhance retina transduction after either systemic or intravitreous injection of rAAV2, -8, or -9 (Petrs-Silva et al., 2008; Zhong et al., 2008; Dalkara et al., 2011). Similarly, the group of Aravind Asokan used random mutagenesis to identify two rAAV9 variants carrying one (N498I) or two (N498Y and L602F) mutations associated with a 10-fold decreased liver transduction without affecting transduction of other organs after a tail vein injection (Pulicherla et al., 2011). Earlier work reported that rAAV2 capsid could sustain insertional mutagenesis without affecting infectivity (Rabinowitz et al., 1999). In this context, peptides derived from a glutamatergic receptor antagonist and dynein-binding motif have been inserted in the VP3 sequence allowing delivery and retrograde transport of rAAV2 to the CNS after peripheral (tongue) injection *in vivo* (Xu et al., 2005). Several groups inserted peptide motifs into rAAV2 capsid after random library or phage-display library screening to increase rAAV2 affinity for coronary or cerebral endothelium (Müller et al., 2003; Chen et al., 2009). Although very promising, further work is necessary to characterize such peptides for brain transduction. DNA shuffling and directed evolution are other methods used to generate mixtures of AAV capsid genes in an unbiased way. Several methods have now been described, but they are all based on a two-step strategy (Maheshri et al., 2006; Gray et al., 2010; Dalkara et al., 2013). First, a library is created by error-prone polymerase chain reaction to induce random mutagenesis on capsid genes or by a combination of different serotypes or random insertion libraries. Second, the library is subjected to several rounds (often three) of selection. One or more clones are obtained with unique characteristics. The Samulski’s group used this method to create clones that can selectively cross the seizure-compromised BBB and transduce specific cells at the damage sites without transducing other organs (Gray et al., 2010). The new vectors generated by directed evolution or a similar strategy might improve neuronal transduction in adult and also overcome the seropositivity problem, since no antibody can be generated for these new capsid variants.

Several methods, not directly relying on the virus, have been proposed to increase brain expression after systemic delivery of rAAV to adult animals. Mannitol has been used to induce hyperosmotic breaching of the BBB, therefore increasing the entry of AAV into the brain (Fu et al., 2011). Although positive results were reported for rAAV2 in a mouse model of LSD, co-administration of mannitol with rAAV9 had only modest effects on brain transduction (McCarty et al., 2009; Fu et al., 2011; Gray et al., 2011b). Those studies suggest that rAAV9 crosses the BBB through active transport (Gray et al., 2011b). Moreover, mannitol co-administration could be risky since it increases the influx of all molecules in the brain. However, this compound is regularly used in clinical practice and no adverse effects have ever been reported in clinical trials (Kaplitt et al., 2007; Lowenstein, 2009). Identification and modification of the key components of such transport might allow transduction improvements. As stated earlier, preexisting anti-AAV antibodies in primate might represent a major obstacle to AAV-mediated gene therapy success. To overcome this concern, Katherine A. High’s group developed an empty mutant capsid, which can interact with antibodies without entering the cell (Mingozzi et al., 2013). As a trojan, addition of the mutant capsid at different ratios in the whole vector formulation increased transduction with the same vector genome dose in both mouse and NHPs (Mingozzi et al., 2013). While safety and efficacy have been proved, this encouraging concept still needs to be tested in a disease model.

## CONCLUDING REMARKS

Ultimately, gene therapy has been a long sought goal for neurological disorder. Thirty-two years after the development of the first recombinant virus, AAV appears to be an extremely useful and promising lynchpin for both therapeutic approaches to neurodegenerative disorders and useful strategies in neuroscientific research. Recent findings demonstrated that several serotypes, such as AAV9 or AAVrh10, cross the BBB with a safe systemic delivery protocol associated with strong non-focal brain transduction. Such a strategy has been proved efficient in several animal models of disease such as SMA or LSDs. Moreover, several innovative strategies, at the genome or capsid levels, have been developed to increase and/or precise tissue-specific gene expression after systemic injection of rAAV. Unavoidable efforts need to be done to harmonize production, purification, titration, and injection protocols between laboratories. It is worth noting that strengthening those specific points will help achieve a clear comparison between these studies. The advancements in AAV-mediated gene therapy hold the promise of a bright future for neurodegenerative diseases.

## Conflict of Interest Statement

The authors declare that the research was conducted in the absence of any commercial or financial relationships that could be construed as a potential conflict of interest.
